# Application of dose-volume histogram prediction in biologically related models for nasopharyngeal carcinomas treatment planning

**DOI:** 10.1186/s13014-020-01623-2

**Published:** 2020-09-15

**Authors:** Wufei Cao, Yongdong Zhuang, Lixin Chen, Xiaowei Liu

**Affiliations:** 1 School of Physics, Sun Yat-sen University, Guangzhou, 510275 China; 2 State Key Laboratory of Oncology in South China, Sun Yat-sen University Cancer Center, Guangzhou, 510060 China; 3 National Cancer Center, Cancer Hospital & Shenzhen Hospital, Chinese Academy of Medical Sciences and Peking Union Medical College, Shenzhen, 518116 China

**Keywords:** DVH prediction, Biologically related models, Nasopharyngeal carcinoma

## Abstract

**Purpose:**

In this study, we employed a gated recurrent unit (GRU)-based recurrent neural network (RNN) using dosimetric information induced by individual beam to predict the dose-volume histogram (DVH) and investigated the feasibility and usefulness of this method in biologically related models for nasopharyngeal carcinomas (NPC) treatment planning.

**Methods and materials:**

One hundred patients with NPC undergoing volumetric modulated arc therapy (VMAT) between 2018 and 2019 were randomly selected for this study. All the VMAT plans were created using the Monaco treatment planning system (Elekta, Sweden) and clinically approved: > 98% of PGTVnx received the prescribed doses of 70 Gy, > 98% of PGTVnd received the prescribed doses of 66 Gy and > 98% of PCTV received 60 Gy. Of these, the data from 80 patients were used to train the GRU-RNN, and the data from the other 20 patients were used for testing. For each NPC patient, the DVHs of different organs at risk were predicted by a trained GRU-based RNN using the information given by individual conformal beams. Based on the predicted DVHs, the equivalent uniform doses (EUD) were calculated and applied as dose constraints during treatment planning optimization. The regenerated VMAT experimental plans (EPs) were evaluated by comparing them with the clinical plans (CPs).

**Results:**

For the 20 test patients, the regenerated EPs guided by the GRU-RNN predictive model achieved good consistency relative to the CPs. The EPs showed better consistency in PTV dose distribution and better dose sparing for many organs at risk, and significant differences were found in the maximum/mean doses to the brainstem, brainstem PRV, spinal cord, lenses, temporal lobes, parotid glands and larynx with *P*-values < 0.05. On average, compared with the CPs, the maximum/mean doses to these OARs were altered by − 3.44 Gy, − 1.94 Gy, − 1.88 Gy, 0.44 Gy, 1.98 Gy, − 1.82 Gy and 2.27 Gy, respectively. In addition, significant differences were also found in brainstem and spinal cord for the dose received by 1 cc volume with 4.11 and 1.67 Gy dose reduction in EPs on average.

**Conclusion:**

The GRU-RNN-based DVH prediction method was capable of accurate DVH prediction. The regenerated plans guided by the predicted EUDs were not inferior to the manual plans, had better consistency in PTVs and better dose sparing in critical OARs, indicating the usefulness and effectiveness of biologically related model in knowledge-based planning.

## Introduction

### Research background and purpose

Intensity-modulated radiation therapy (IMRT) and volumetric modulated arc therapy (VMAT) allow increased conformity of high-radiation-dose regions to the planning target volume (PTV) while sparing each organ at risk (OAR) [1]. In recent years, a number of efforts to aid in treatment planning using knowledge-based planning (KBP) techniques have improved the consistency of plan quality and reduced the required optimization time. Most of these efforts were developed based on establishing a correlation between the OAR-PTV anatomy and the OAR cumulative dose-volume histogram (DVH). The most popular tools for quantifying the OAR-PTV anatomy, namely, the overlap volume histogram (OVH) [2, 3] and the distance-to-target histogram (DTH) [4, 5], were equivalent when the Euclidean distance function was used in the DTH. However, one concern regarding the DTH and OVH is that their simplicity may lead to inaccurate presentation of the interpatient variations in anatomical features, which may have an impact on the organ dose deposition [5, 6], especially for complex tumour volumes in close proximity to critical structures such as those observed in nasopharyngeal carcinomas (NPCs). The dose deposited in an OAR voxel depends not only on its distance from the PTV surface but also on the treatment beam orientation [5, 7, 8].

Recent studies indicated that using dosimetric features might be a new avenue for research and development [8–10]. Ming Ma [8, 9] used PTV-only patient treatment plans to estimate their potentially achievable quality using dosimetric parameters as model input. Their results demonstrate the potential of DVH and 3D dose distribution prediction based on dosimetric information. In our previous work [10], we employed dosimetric information resulting from individual conformal beams in different directions to predict the DVHs. The results showed that this method was of great accuracy in prediction and great effectiveness in treatment planning.

Defining the dose constraints in reverse optimization is highly important in DVH prediction-based planning. Usually, the planner defines physical dose constraints for each structure of the treatment plan, either in the form of minimum and maximum doses or as dose-volume constraints. Many studies have also reported optimization methods based on biological effects, such as the EUD (equivalent uniform dose) [11–14]. In this study, we follow the method proposed in our previous work [10] and predict the DVH achieved with VMAT. Based on the predicted DVHs, the EUD was calculated and directly applied as OAR dose constraints in biologically related models.

## Methods and materials

**Fig. 1:**
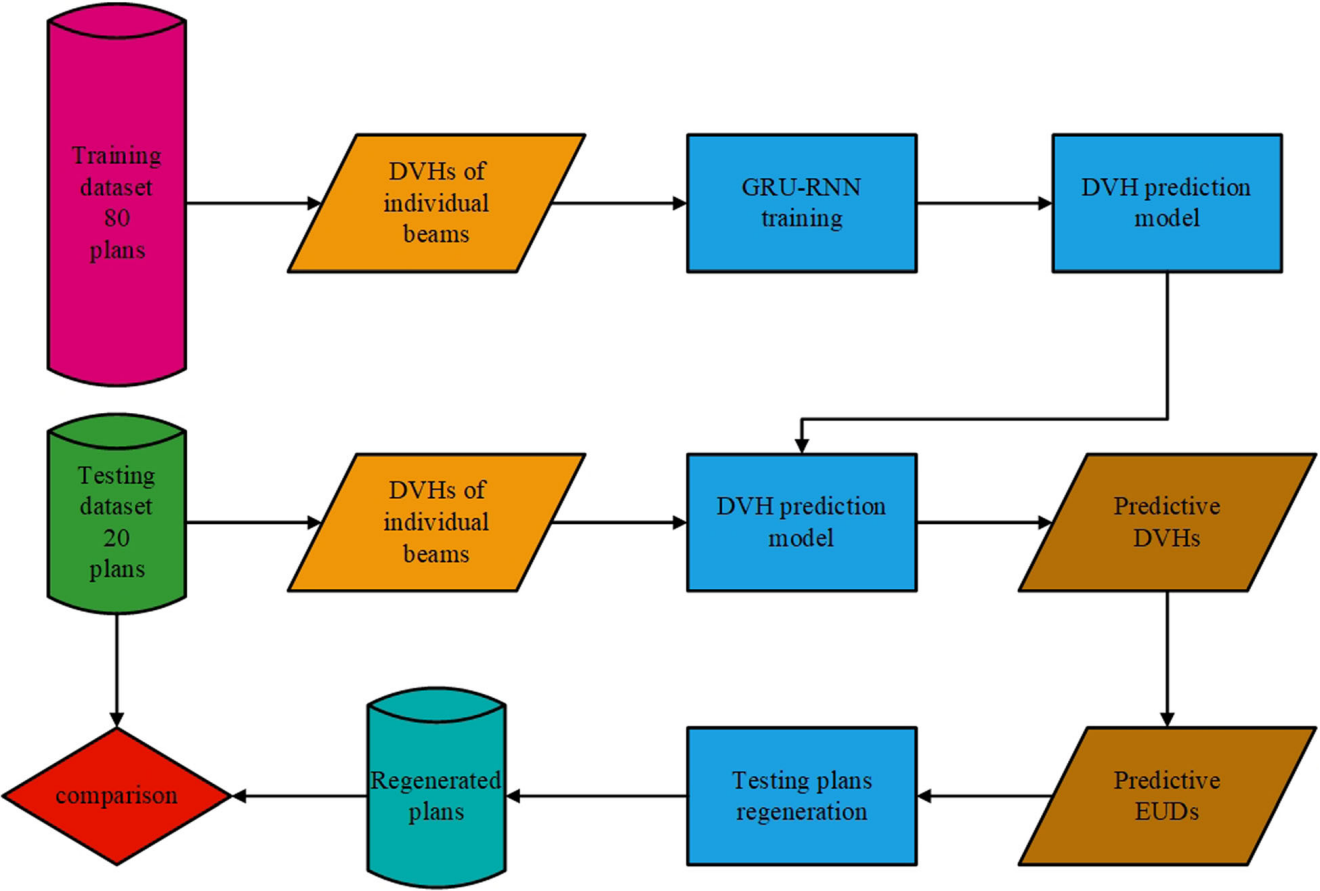
Flowchart showing the individual beams information driven DVH prediction and the predictive EUD based planning process

In this work, a gated recurrent unit-based recurrent neural network (GRU-RNN) based primarily on our previous work [10] was employed for DVH prediction. The DVHs for a certain OAR induced by 9 different individual conformal beams with equal angle intervals were used as GRU-RNN inputs. The corresponding DVH of the treated plan of this given OAR was used as the desired output. Based on the trained model, the DVHs were predicted for other cases, and the regenerated plans driven by the EUDs calculated from the predicted DVHs were compared with the treated plan. A flowchart of the individual beam information-driven DVH prediction and the predictive EUD-based planning processes is shown in Fig. 1.

### Data acquisition

**Fig. 2:**
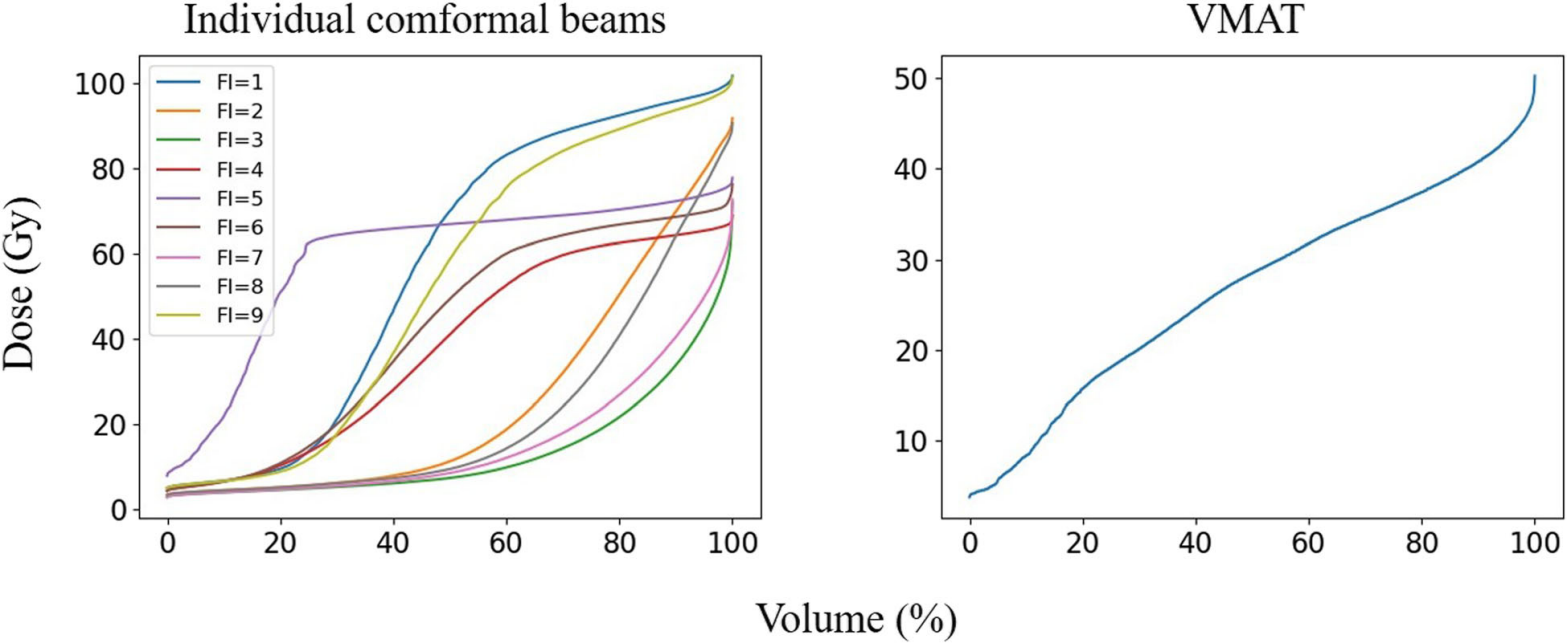
An example of the DVHs for a patient’s brainstem, the gantry angles of which denotes as FI = 1,2,3, ...,9, were 160, 120, 80, 40, 0, 320, 280, 240, and 200 degrees, respectively. Dose (Gy) and Volume (%) represent the delivered dose and percent OAR volume, respectively

One hundred patients with NPC undergoing volumetric modulated arc therapy (VMAT) between 2018 and 2019 were randomly selected for this study. Following ICRU-83 report [15], radiation oncologists delineated the gross tumour volume of the nasopharynx (GTVnx), the gross tumour volume of the metastatic lymph node (GTVnd), the clinical target volume (CTV), and the OARs in the planning CT. A margin of 3 mm was applied around the GTVnx, GTVnd and CTV to create the planning GTV, PGTVnx and PGTVnd, and planning target volume (PTV), respectively. All the VMAT plans were created using the Monaco treatment planning system (Elekta, Sweden, V1.0, 2013) and clinically approved: > 98% of PGTVnx received the prescribed doses of 70 Gy, > 98% of PGTVnd received 60 Gy, and > 98% of PTV received 60 Gy. In addition to the approved plans, a nine-field PTV-conforming radiotherapy plan was generated, resulting in just 98% of PGTVnx receiving the prescribed doses of 70 Gy. The DVHs of a patient’s brainstem induced by 9 different individual conformal beams and the DVH of the given patient’s brainstem from the VMAT plan are shown in Fig. 2. Different from our previous work [10], the DVHs were resampled by volume bin by percentage (0.1% in practice) rather than by absolute volume or dose values, making the DVHs of equal length, as shown in Fig. 2. The OARs considered during training included the brainstem, spinal cord, optic chiasm, optic nerves, lens, parotid glands (excluding the overlap with PTVs), larynx (excluding the overlap with PTVs), temporal lobes (excluding the overlap with PTVs) and the planning organ-at-risk volumes (PRVs).

### DVH prediction

**Fig. 3:**
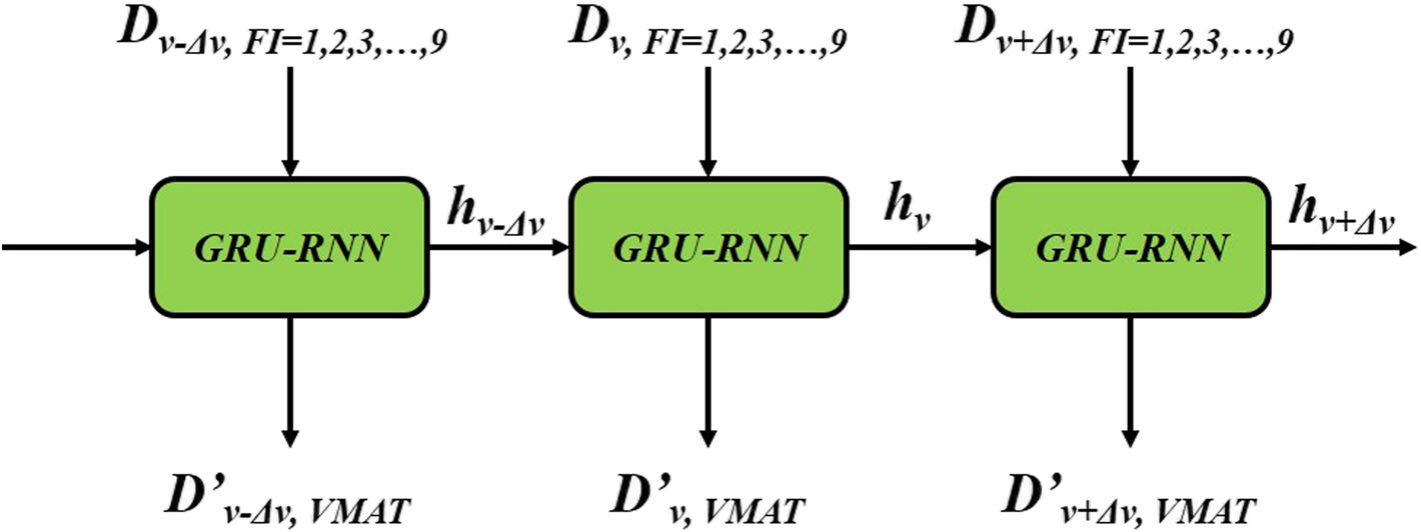
Flow chart of the GRU-RNN. RNN-GRU consists of 3 GRUs with the sizes of hidden states were 18, 9 and 1, respectively. *D*_*v*_ mean the volume proportion with deposition not greater than D was v and ${D}_v^{\prime }$ was the predictive value. In the practical, *Δv* was set to 0.1%

The GRU-based RNN prediction model in this study was established using the PyTorch (Facebook, US) framework, as shown in Fig. 3. *D*_*v*_ in Fig. 3 shows that a ≤ D Gy dose was delivered to v of the OAR volume. The GRU-RNN was trained by the Adam optimizer with the goal of minimizing the MSE, defined as follows:

1 $$ MSE={\sum}_{P=1}^{80}{\sum}^{OARs}{\left\Vert DV{H}_{P, OAR}^{\prime }- DV{H}_{P, OAR}\right\Vert}^2 $$

where P refers to a patient whose plan participated in the training process, DVH′ is the predicted DVH and DVH is the actual DVH. Because the EUD constraints were directly applied as dose constraints in the OAR optimizations, the *δ*_*OAR*_ and *σ*_*OAR*_ of the EUD were used to evaluate the prediction accuracy and precision of a given OAR with the trained GRU-RNN. In this study, *δ*_*OAR*_ and *σ*_*OAR*_ were defined as follows:

2 $$ EU{D}_{OAR}={\left({\sum}_{\mathrm{v}=0}^{\mathrm{v}=1}\left(\Delta  v\bullet {D}_v^k\right)\right)}^{\frac{1}{k}} $$

3 $$ {\delta}_{P, OAR}=\mid {EUD}_{P, OAR}^{\prime }- EU{D}_{P, OAR}\mid $$

4 $$ {\delta}_{OAR}=\frac{\sum_{P=1}^{20}{\delta}_{P, OAR}}{n} $$

5 $$ {\sigma}_{OAR}=\sqrt{\frac{\sum_{P=1}^{20}{\left({\delta}_{P, OAR}-{\delta}_{OAR}\right)}^2}{n-1}} $$

where, *k* is the power law exponent. In practice, *k* was set to 10.0 for the spinal cord, 9.8 for the brainstem, optic nerves, and optic chiasm, 6.8 for the larynx, 2.0 for the lens and 3.9 for the parotid glands. EUD′ denotes the EUD calculated based on the predicted DVH, while EUD was calculated based on the actual DVH. An OAR and its corresponding PRV share the same *k* value. Following the Monaco planning protocol, we choose *k* = 0.15 × *D*_50_ for different OARs. The *D*_50_ [16] value is the dose that causes a complication in 50% of all patients.

### Experimental plan based on predicted DVH

For 20 test patients, the EUD values calculated based on the predicted DVHs were applied as dose constraints for VMAT optimization. In this work, a corrected EUD constraint, c*EUD*′_*OAR*_, was applied during optimization. A 0 cm shrink margin was applied to the parotids, larynx and temporal lobes to exclude overlaps with the PTVs. For a certain OAR:

6 $$ cEUD{\prime}_{OAR}=\alpha \bullet {c}_{OAR}\bullet EUD{\prime}_{OAR} $$

7 $$ {c}_{OAR}=\frac{1}{80}{\sum}_{P=1}^{80}\left(\frac{EU{D}_{P, OAR}}{ EU D{\prime}_{P, OAR}}\right) $$

where *c*_*OAR*_ was used to correct the prediction values for different OARs based on the training dataset. α was set slightly smaller than 1 to achieve stricter constraints, 0.97 in practice. A corrected maximum dose constraint calculated with a similar process was also considered for the spinal cord and lens. To investigate the feasibility and usefulness of this method without the need for manual touch-up during VMAT optimization, the optimization procedure was executed only once.

## Results

### EUD prediction results

**Fig. 4:**
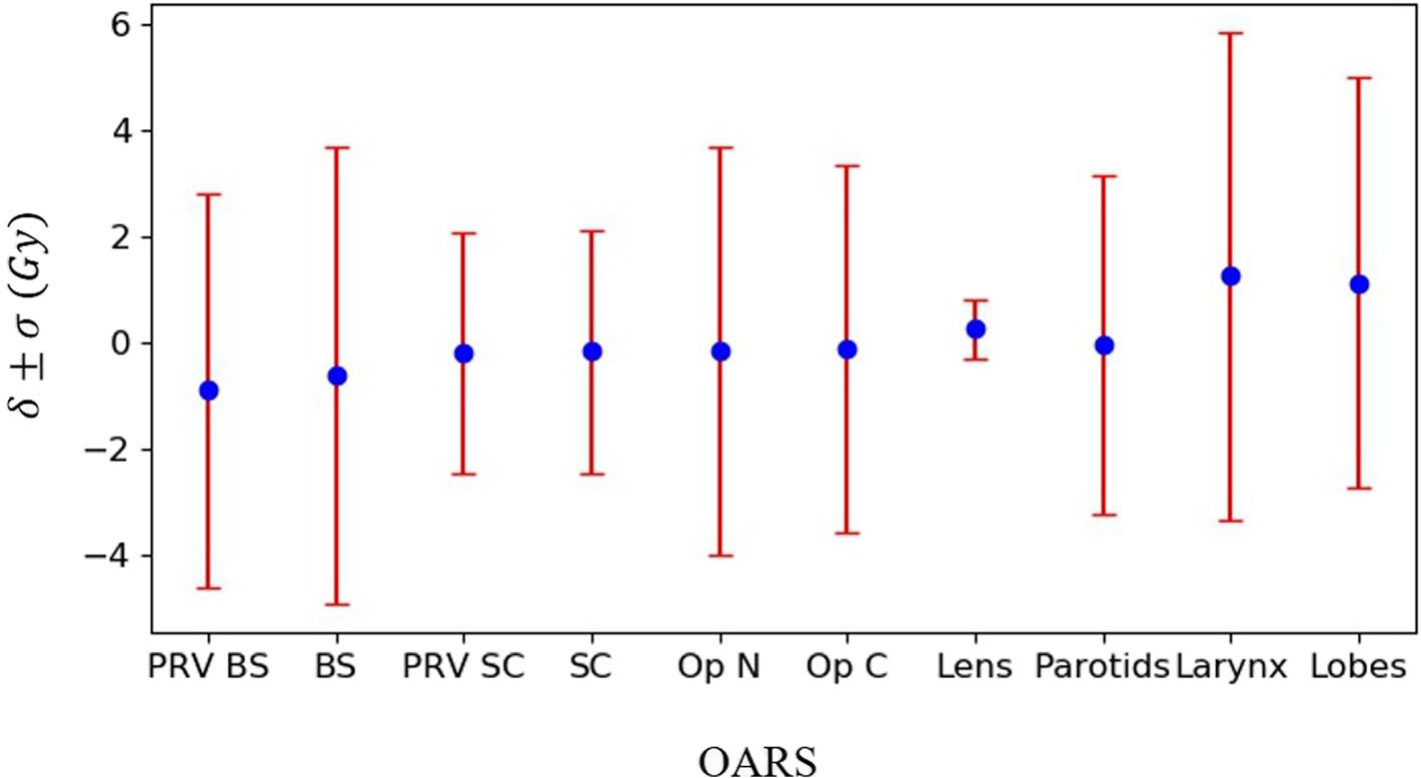
The δ and σ presented the maximum dose prediction accuracy and precision of different OARs, including the brainstem (BS), spinal cord (SC), optic nerves (Op N), optic chiasm (Op C), lens, parotid glands (Parotids), larynx, and temporal lobes (Lobes)

Figure 4 shows the prediction accuracy and precision of the EUD for the different OARs of the 20 testing patients. The results show that the GRU-RNN achieved good prediction accuracy for all OARs and its performances on training and test patients were quite close. For the predicted EUDs of the testing patients, the parotid glands had the smallest δ of − 0.04 Gy, with a σ of 3.19 Gy; except for the lens with a σ of 0.56 Gy, the spinal cord and its PRV had the smallest σ values of 2.28 and 2.27 Gy, with δ values of − 0.17 and − 0.18 Gy, respectively, and the larynx had the largest δ (1.25 Gy) and the largest σ (4.58 Gy).

### Experimental plans VS clinical plans

To conduct the comparisons, we used Wilcoxon signed rank tests to compare the dosimetric results among the 20 testing patients between the clinical plans (CPs) and the experimental plan (EPs), which were regenerated based on the predicted EUDs. Differences were considered statistically significant at *P* < 0.05. Table 1 provides a summary of the dosimetric results comparisons for the test patients between the CPs and EPs. No significant differences were found for the PTVs with the mean difference of D_98_ was less than 0.1 Gy. For the OARs, significant differences were found in the maximum/mean doses to the brainstem, brainstem PRV, spinal cord, lenses, temporal lobes, parotid glands and larynx with *P*-values < 0.05. On average, compared with the CPs, the maximum/mean doses to these OARs were altered by − 3.44 Gy, − 1.94 Gy, − 1.88 Gy, 0.44 Gy, 1.98 Gy, − 1.82 Gy and 2.27 Gy, respectively. In addition, significant differences were also found in brainstem and spinal cord for the dose received by 1 cc volume with 4.11 and 1.67 Gy dose reduction on average.

**Table 1:** Summary of the average±standard deviation dosimetric results

Structure	Criterion	CPs	EPs	***p***-value
PTV70	*D*_*98*_	70.71 ± 0.83	70.77 ± 0.28	0.794
PTV66	*D*_*98*_	66.42 ± 0.89	66.52 ± 0.66	0.612
PTV60	*D*_*98*_	62.25 ± 1.60	62.17 ± 1.30	0.911
Brain Stem	*D*_*max*_*, Gy*	58.20 ± 5.21	54.76 ± 4.85	**< 0.01**
	*D*_*1cc*_*,Gy*	50.07 ± 4.37	45.96 ± 4.72	**< 0.01**
Brain Stem PRV	*D*_*max*_*, Gy*	62.46 ± 5.65	60.52 ± 5.24	**0.048**
Spinal Cord	*D*_*max*_*, Gy*	41.33 ± 2.38	39.45 ± 1.15	**< 0.01**
	*D*_*1cc*_*,Gy*	38.06 ± 2.25	36.39 ± 1.07	**0.01**
Spinal Cord PRV	*D*_*max*_*, Gy*	46.75 ± 1.98	46.41 ± 3.17	0.601
Optic Chiasm	*D*_*max*_*, Gy*	39.60 ± 19.65	38.47 ± 18.76	0.379
Lens*	*D*_*max*_*, Gy*	6.93 ± 2.03	7.37 ± 2.01	**0.044**
Optic Nerves*	*D*_*max*_*, Gy*	47.77 ± 15.90	47.17 ± 15.89	0.657
TP Lobes*	*D*_*max*_*, Gy*	68.30 ± 6.65	70.28 ± 5.04	**0.034**
Parotids*	*D*_*mean*_*, Gy*	37.81 ± 6.60	35.99 ± 6.86	**< 0.01**
Larynx	*D*_*mean*_*, Gy*	44.08 ± 2.55	46.35 ± 2.78	0.087

* denotes the bilateral organs

**Fig. 5:**
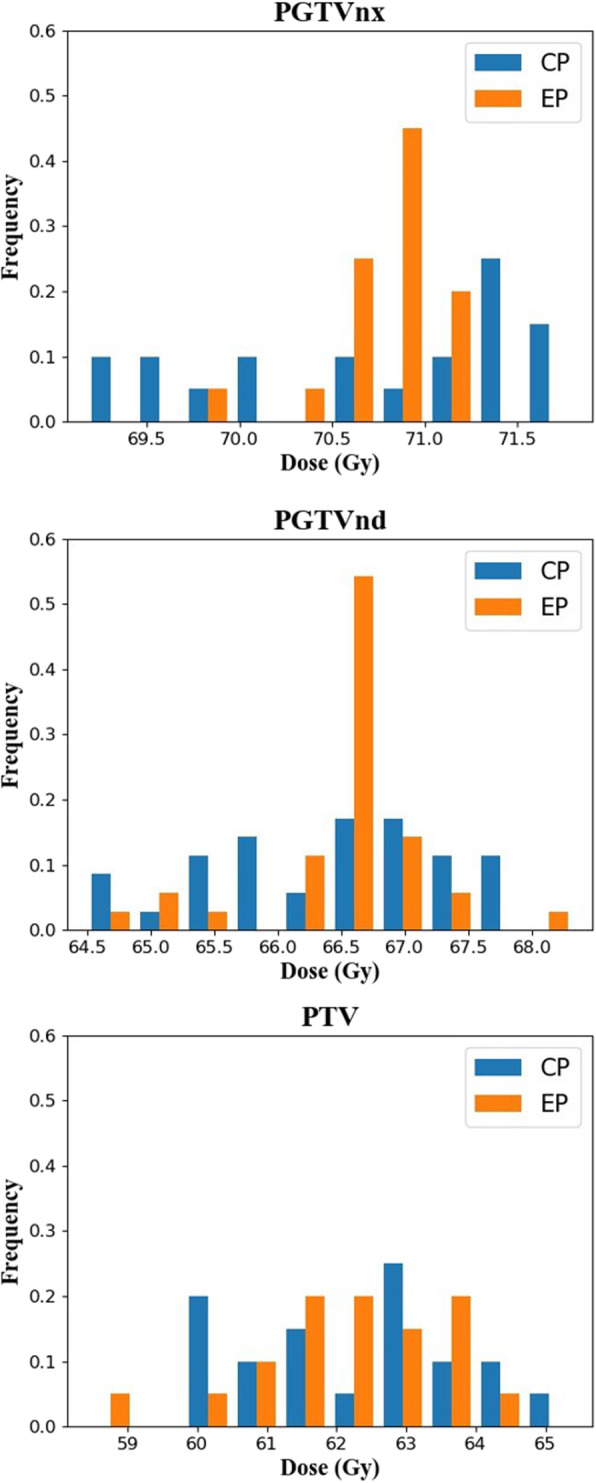
The distribution of D_98_ to the PTVs

Figure 5 displays the distribution of D_98_ in the PTVs from the CPs and EPs. The D_98_ of EPs had notably different distributions from those of the CPs; nearly 60% of the cases of PGTVnx and PGTVnd were located in the range 70.5–71 Gy. The PTV distributions of the CPs and EPs was very close. The distribution of D_98_ for the PTVs was consistent with the standard deviation results in Table 1.

**Fig. 6:**
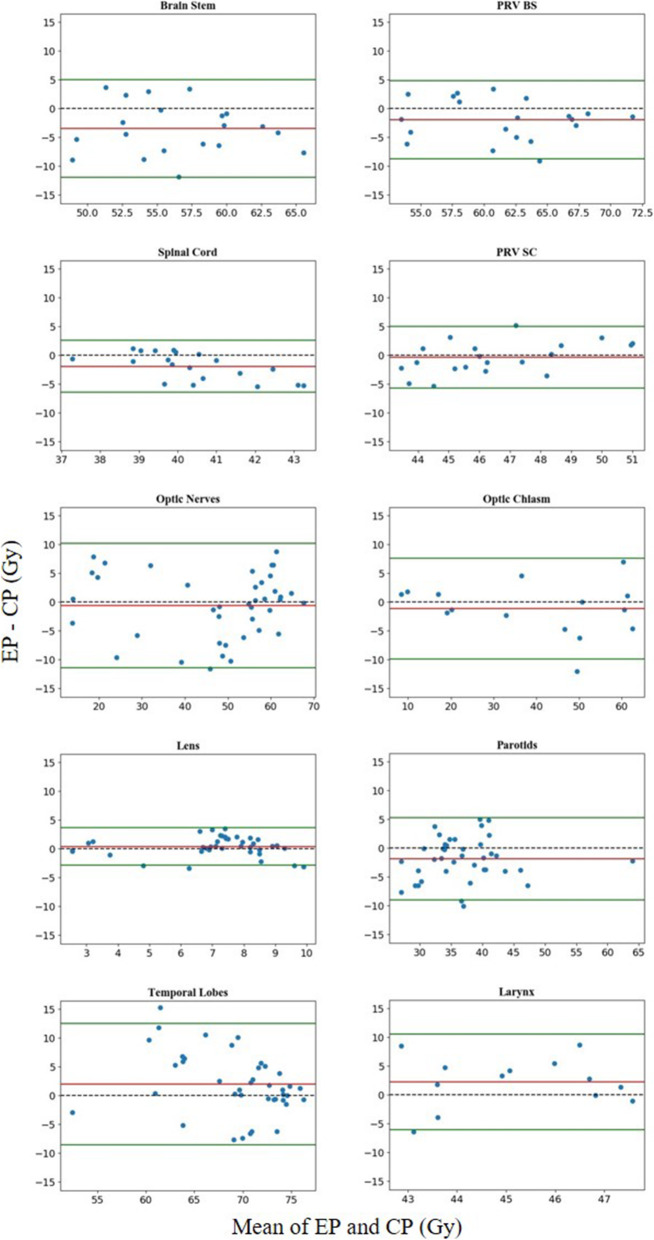
Difference between CPs and EPs. Horizontal lines were drawn at the line of equality (black dashed line), the mean difference (red line), and the limits of agreement (green lines). The limits of agreement were defined as the mean difference ± 1.96 SD of the differences

Figure 6 displays the difference between pairs of EP and CP results for all 20 patients in relation to the mean of this pair of results. The differences for almost all the OARs were located within the limits of agreement at frequencies above 95%, except for lenses, which had a frequency of 90% (38/40). Notable biases in the differences between EPs and CPs were found in the brainstem, spinal cord, lenses, temporal lobes, parotids and larynx. For the brainstem, spinal cord and parotids, patients with lower maximum/mean doses of EPs accounted for 80, 70 and 72.5%, respectively, and the maximum differences were 11.80, 5.40 and 10.04 Gy lower, respectively. For lenses, temporal lobes and larynx, patients with lower maximum/mean dose in EPs accounted for 32.5, 32.5 and 20.0%, and the maximum differences were 3.50, 15.30 and 10.81 Gy.

## Discussion

### GRU-RNN for DVH prediction

**Fig. 7:**
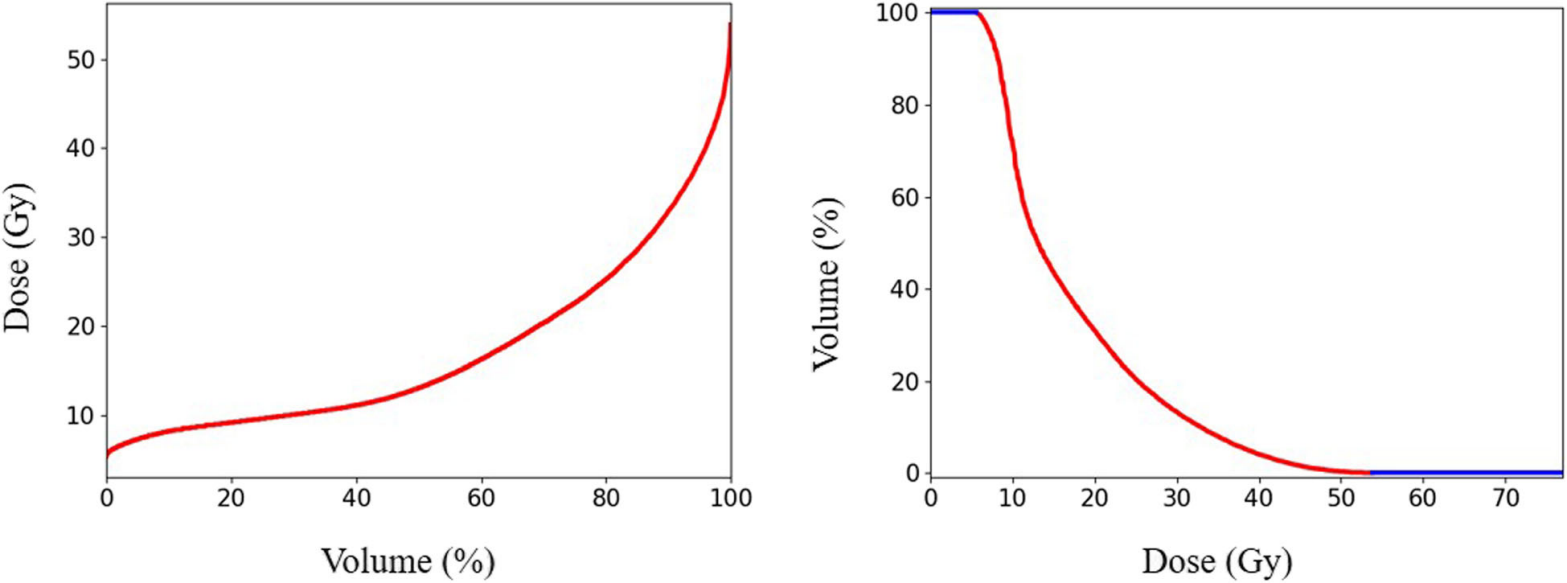
Different DVH forms for GRU-RNN

To process sequential data (DVH, etc.), a regular neural network (such as a fully connected network, convolutional neural network, etc.) could also be suitable but would be limited by the fixed input vector size. RNN and similar models, such as the GRU-RNN used in this study, are particularly suitable for predicting the entire DVH rather than only fixed amount of interesting points. Figure 7 shows the different DVH forms adopted in this work and the previous work [10]. We found that the contributions of MSE in Eq. (1) majorly came from the effective region (red line in Fig. 7) in DVH. Percentage dose bin (0.1% in practice), rather than by absolute volume or dose values, helped focusing the neural network training attention on the effective region, which was shown to be helpful in improving the prediction accuracy through practical experiments. Besides, making the effective part of equal length could also help balanced the weights of different OARs in training process. The relationships between the DVHs induced by individual beams and the DVH of the treated VMAT plan may be related to the potential for the TPS to optimize the beamlet intensity or ray flux to meet the clinical dose-volume constraints. The EUD prediction result further confirms the feasibility and applicability of using individual beam dosimetric information for DVH prediction. In addition, the OAR-specific corrected parameter, c_OAR_ in Eq. (7), was also helpful in improving the prediction accuracy of the OAR-independent GRU-RNN.

### Biologically related models for treatment planning

EUD constraints, rather than physical dose constraints, were employed for the inverse radiotherapy planning in this study. Compared to the CPs, better consistency was achieved for the PTVs in the EPs; most were just above the prescription requirements, especially for PGTVnx. In addition, better dose sparing was also achieved for most of the OARs in the EPs, especially for critical OARs such as the brainstem and spinal cord. The dosimetric results were further improved compared to our previous work [10], which may be mainly owe to the EUD-based objectives allowing exploration of a much larger universe of solutions, making it easier for the optimization system to balance competing requirements in search of a better solution [13]. Besides, α in Eq. (6) also had certain affect, which was found in the trial and error process. The results not only indicated the usefulness and effectiveness of the proposed method in treatment planning but also demonstrates the advantage of using biologically related models in treatment planning. In addition, biologically related models offer an easier way to convert clinical intent to DVH-based objectives, such as the EUD, which is of significant benefits in knowledge-based planning. As shown in Table 1, not all the OARs achieved better dose sparing, such as the temporal lobes and larynx. This might be caused by the setting of the power law exponent, k. In treatment planning, we noticed that in some of the clinical plans, larger k values were used for these OARs, which would lead to assigning greater weights to the maximum dose constraint. Finding a more flexible and individualized k value might be worth further research in biologically related models for treatment planning.

In our preliminary experiment, when all the OARs of the parotid glands, larynx and temporal lobes were used in training and practical application, the dose constraints did not achieve the desired results. Combined with our clinical experience, partial OARs, excluding the overlap with PTVs rather than the complete OARs, were considered in training. For practical application, a 0 cm shrink margin was applied to the dose constraints during optimization. The results show that this method achieves the desired effect and indicates the advantages of separating the OAR tissue inside and outside the PTV region during treatment planning.

## Conclusion

The GRU-RNN-based DVH prediction method was capable of accurate DVH prediction. The regenerated plans guided by the predicted EUDs were not inferior to the manual plans, had better consistency in PTVs and better dose sparing in critical OARs, indicating the usefulness and effectiveness of biologically related model in knowledge-based planning.

## Data Availability

All data generated or analysed during this study were included in this published article.
